# Perspectives on Heavy Metal Soil Testing Among Community Gardeners in the United States: A Mixed Methods Approach

**DOI:** 10.3390/ijerph16132350

**Published:** 2019-07-03

**Authors:** Candis M. Hunter, Dana H. Z. Williamson, Matthew O. Gribble, Halle Bradshaw, Melanie Pearson, Eri Saikawa, P. Barry Ryan, Michelle Kegler

**Affiliations:** 1 Department of Environmental Health, Emory University, 1518 Clifton Rd. Atlanta, GA 30322, USA; 2 Department of Behavioral Health Sciences and Education, Emory University, 1518 Clifton Rd. Atlanta, GA 30322, USA; 3 Department of Epidemiology, Emory University, 1518 Clifton Rd. Atlanta, GA 30322, USA; 4 Department of Environmental Sciences, Emory University, 400 Dowman Drive, Atlanta, GA 30322, USA

**Keywords:** soil contaminants, urban agriculture, environmental psychology, risk perception, Theory of Planned Behavior

## Abstract

Community gardens offer numerous benefits, but there are also potential risks from exposure to chemical contaminants in the soil. Through the lens of the Theory of Planned Behavior, this mixed methods study examined community gardeners’ beliefs and intentions to conduct heavy metal soil testing. The qualitative component involved five focus groups of community garden leaders in Atlanta, Georgia. Qualitative analysis of the focus group data revealed that heavy metal soil contamination was not frequently identified as a common gardening hazard and several barriers limited soil testing in community gardens. The focus group results informed the development of a questionnaire that was administered to 500 community gardeners across the United States. Logistic regression analysis revealed that the soil testing intention was associated with attitude (aOR = 2.46, 95% CI: 1.34, 4.53), subjective norms (aOR = 3.39 95% CI: 2.07, 5.57), and perceived behavioral control (aOR = 1.81, 95% CI: 1.10, 2.99). Study findings have implications for interventions involving community garden risk mitigation, particularly gardens that engage children and vulnerable populations.

## 1. Introduction

Community gardens are growing in popularity and are associated with an array of positive outcomes [1,2,3]. The American Community Gardening Association estimates that there are over 18,000 community gardens in at least 250 towns in the United States and Canada [2]. Practitioners and academics from public health, urban planning, education, environmental management, and sustainability sectors investigate and promote community garden benefits [4,5]. For example, the Centers for Disease Control and Prevention recommends community gardens as a strategic priority to improve fruit and vegetable consumption [6], and the American Planning Association advocates for the utility of community gardens in food systems planning [7]. 

Heavy metals are naturally-occurring, high density elements with widespread accumulation and non-biodegradability in soils [8]. Based on their toxicity and potential for human exposure, heavy metals such as lead and cadmium are ranked among the top 10 hazardous substances of public health concern by the Agency for Toxic Substances and Disease Registry and the World Health Organization [9,10]. Plant species, soil composition, and several other factors affect whether plants are able to absorb toxic heavy metals [10,11]. Due to natural processes and anthropogenic sources of pollution in urban environments, toxic heavy metals may be present in urban community garden soils [12,13,14]. For example, past and current activities including construction, manufacturing, pesticide application, landfill waste burial, and lead paint and gasoline disposal are contributors to heavy metal contamination in urban soils [15,16]. Therefore, soil testing is often recommended as a best management practice in urban community gardens [17,18,19,20]. Several studies have quantified soil contaminant concentrations in urban gardens [13,21,22,23,24,25,26]; however, understanding gardeners’ beliefs and perceptions are critical to the development of interventions that abate these risks. 

Only a few studies have examined soil contamination testing and other practices to reduce soil toxicant exposures among community gardeners in the United States. Most U.S. studies on gardeners’ attitudes and knowledge related to soil contamination typically have small sample sizes and were conducted in states with large urban cities in the Northeast, Midwest and West. These studies have documented that challenges to soil testing include cost, sampling uncertainty, contaminant spatial variability, interpretation of results, and lack of clear guidelines/screening levels for some metals [27,28,29]. Moreover, these studies indicate that community gardeners in urban environments exhibit varied knowledge and concerns about soil contaminants, site history, and methods to reduce exposure to soil contaminants. 

To understand community gardeners’ views and knowledge of soil contamination, researchers administered surveys to 70 gardeners and 18 community garden key informants in Baltimore, Maryland. Findings from this research suggested that gardeners were often unaware of some soil contaminants and methods of reducing exposure. Additionally, barriers such as cost and complicated processing deterred gardeners from testing their soil and identifying the site history [27]. A study of urban gardeners in Missouri and Washington, revealed that over 50% of the respondents were unaware of how to detect the presence of soil contaminants in an urban environment and where to send their soils for sampling [30]. Another survey of 20 community gardens in Missouri indicated that most gardeners were not concerned about soil contamination, but Black or African American gardeners, in particular, were more likely to be concerned about soil contamination [31]. Focus group findings from an urban agricultural study in Ohio among low-income residents conveyed a need for more information about soil quality, soil testing, remediation, and garden startup costs [32]. Semi-structured interviews of 17 community gardeners in the Boston area revealed that gardeners had knowledge and interest in soil testing, but about half were unable to test their soil due to soil testing costs and other concerns [29]. 

The Theory of Planned Behavior (TPB) provides a well-established conceptual framework for understanding behaviors and designing behavioral interventions [33]. According to TPB, attitude towards a behavior, subjective norms, and perceived behavioral control are the primary influencers of behavioral intention, which is an antecedent to behavior [34,35]. Attitude toward the behavior is the extent to which the behavior is favorably or unfavorably valued. Behavioral beliefs are underlying convictions that the behavior will produce a given outcome (e.g., soil testing will detect potential soil problems; soil testing will result in liability concerns), and behavioral beliefs precede the attitude toward the behavior. Subjective norms are perceived social pressures to perform or not perform a behavior. Normative beliefs are perceived behavioral expectations of referents (e.g., other gardeners, environmental organizations) and serve as an antecedent to subjective norms. Perceived behavioral control (PBC) is assumed ability to conduct a behavior, which is influenced by the presence of conditions that make a behavior easy or difficult to perform. Control beliefs are antecedents of PBC and are beliefs about the presence of factors that may serve as barriers or facilitators to performing the behavior (e.g., soil testing is expensive, soil testing is too complicated). The theory hypothesizes that the stronger PBC and higher favorability of attitudes and subjective norms toward a behavior, the stronger the behavioral intention. Behavioral intention, in turn, is a strong predictor of the behavior, particularly when actual behavioral control is high. 

While not focused on gardens per se, several studies of farmers have demonstrated that attitude and perceived behavioral control, which are constructs from the TPB, can affect their adoption of practices related to food safety, soil management, and other sustainable agricultural practices [36,37,38,39,40]. However, data are limited on whether these findings can be applied to urban community gardeners, who have different motivations and resources than commercial farmers or farmworkers. To effectively evaluate, prioritize, and address soil testing challenges among community gardeners, a behavioral theory approach can reveal psychosocial factors that influence community gardeners’ soil testing perspectives.

Using TPB as a theoretical framework, the aim of this exploratory mixed methods study is to investigate factors that may contribute to heavy metal soil testing among U.S. community gardeners. According to the TPB, it is hypothesized that perceived behavioral control and higher favorability of attitudes and subjective norms will lead to soil testing intention. Demographic and sociocultural variables are often considered background factors in TPB that may also impact beliefs, attitudes, and perceived behavioral control towards a behavior. Therefore, this study also examined gardener demographics as well as garden contextual variables such as garden region location, garden site history, garden chemical practices, and proximity to older housing stock and roadways [41] that may impact soil contaminant concentrations and exposure. Several studies have indicated that past behavior is positively associated with attitudes and behavioral intention [42,43,44,45], so previous soil testing history was also explored. Garden locations and contexts may influence soil testing and other mitigation behaviors, since some cities (e.g., New York, Chicago and Boston) may require garden soil testing and have industrial histories that could contribute to greater soil contamination.

## 2. Materials and Methods 

First, the qualitative phase involved focus groups of Atlanta community garden leaders. Focus group participants were recruited via email solicitation utilizing listservs from community garden and environmental organizations. To be eligible to participate in the focus group, community garden administrators/leaders had to be at least 18 years of age and involved at their present community gardening site in five metropolitan Atlanta counties (Clayton, Cobb, DeKalb, Fulton, and Gwinnett) for at least three months. As an incentive for focus group participation, potential participants were offered a free heavy metal soil screening (valued at $35), interpretation of soil screening results, and a gardening gift consisting of gloves, trowel, seeds, and best practices information on reducing exposure to soil contaminants in urban environments.

To guide the focus group discussion, a semi-structured discussion guide based on TPB guidelines [46] was developed. Participants described salient beliefs related to risk perception of soil contaminants; advantages/disadvantages of soil testing to inform attitude and behavioral beliefs; advocates and opponents of soil testing to inform normative beliefs and subjective norms; barriers and facilitators to soil testing to inform control beliefs and PBC. Focus groups were facilitated and audio recorded by the first author (C.H.). The focus groups were conducted at quiet meeting rooms at Atlanta libraries and county Extension conference rooms. 

Guided by TPB questionnaire development criteria [47], the strongest behavioral, normative, and control beliefs from the focus groups were used to develop the questionnaire items. Initial questionnaire items were piloted among gardeners that were recruited via email from community garden leaders in the Atlanta area and then interviewed to solicit feedback regarding questionnaire length, wording, question sequence, incentives, and overall online usability. Based on the pilot questionnaire respondents’ recommendations, the questionnaire was modified and retested among pilot respondents to create the final questionnaire.

The second phase of the study utilized a cross-sectional, online questionnaire that was open to potential respondents from August to September 2017. The study population was community gardeners and leaders who were at least 18 years old. Participants were initially recruited in person at a recruitment booth during the 2017 American Community Gardening Association Conference (ACGA) in Hartford, CT. Study participants were asked to complete the online questionnaire on an electronic tablet. Hardcopies of the questionnaire were available at the recruitment booth. The research questionnaire link was also shared on the ACGA Facebook page and was subsequently re-shared on Facebook by community garden groups affiliated with ACGA. Community gardeners received an electronic $10 Amazon gift card as an incentive for participating in the questionnaire. The research protocol was reviewed and determined exempt by Emory University Institutional Review Board.

### 2.1. Measures and Description of Questionnaire Questions

The questionnaire included ten sections: knowledge and risk perception of soil contaminants, past gardening behaviors, beliefs related to heavy metal soil testing, heavy metal soil testing subjective norms, heavy metal soil testing barriers and facilitators, heavy metal soil testing intention, handwashing practices, compost practices, community garden information, and demographic information. All TPB questionnaire items were measured using a 5-point Likert Scale with the anchors differing based on the primary TPB construct. Each TPB construct was created as the average of the items according to TPB guidelines [46,47]. Attitudes toward soil testing (A) were calculated as the average of three-items as indicated in Table 1, with response options ranging from 1 (unimportant) to 5 (important). Subjective Norms (SN) were calculated as the average of three-items with ratings ranging from 1 (strongly disagree) to 5 (strongly agree). For example, participants were asked to rank “I feel social pressure to conduct heavy metal soil testing in my community garden plot.” Perceived Behavioral Control (PBC) was calculated as the average by item measures. For example, participants ranked “Heavy metal soil testing in my community garden plot” is 1 (not under my control) to 5 (under my control), to assess PBC. 

To assess Intention to test their soil or wash their hands, participants were asked to rate three statements such as “I expect to conduct heavy metal soil testing at my garden plot during the next growing season” from (1) strongly disagree to (5) strongly agree. These items were averaged to create an Intention score. Intention scores in the study were highly skewed with over 70% participants indicating that they either agreed or strongly agreed that they were likely to conduct heavy metal soil testing. Since logarithm and other types of transformations did not improve the overall distribution of the intention variables, these variables were dichotomized where responses of 1 to less than 3.5 were coded as “Low Intention”, and responses greater than 3.5 were coded as “High Intention” [48].

### 2.2. Data Analysis

Focus group recordings were transcribed verbatim, and a code book was developed based on TPB research questions (deductive codes) and salient themes from the focus group transcripts (inductive codes). Two analysts independently reviewed and coded the focus group transcripts. Intercoder agreement was established by comparing the independently coded transcripts and resolving any coding discrepancies through discussion. The coded themes (nodes) were entered into NViVo 10 (QSR International Pty Ltd., Melbourne, Australia), and content analysis was performed using NVivo summary reports by Node output to identify themes and patterns based on the primary research questions. Primary themes and summaries were based on frequency of mentions and consistency across focus groups. 

All questionnaire data were imported into Statistics and Data Analysis (Stata 15.0, StataCorp LP, College Station, TX, USA) software, cleaned, and coded appropriately (e.g., reverse coded, dummy variables where applicable). TPB variables were screened for missing data, normality, collinearity, and correlations among variables. Descriptive statistics, including means, standard deviations, and correlations were calculated and examined for questionnaire items underlying each TPB construct. Since questionnaire items are novel and have not been previously reported in this study population, Cronbach’s alpha was calculated to assess internal consistency among TPB items. Frequencies were used to examine distributions. Chi-square tests were used to determine if the intention of each behavior was significantly associated with demographic variables. Logistic regression with robust standard errors to account for potential social connections among gardeners was used to predict soil testing intention with TPB predictors [49]. Demographic and garden contextual variables that were significantly associated with intention were included in the logistic regression analysis as covariables. These variables were screened for intervariable correlation prior to inclusion in the logistic regression. For all statistical tests, statistical significance was defined by *p*-values <0.05.

Of the 500 questionnaire respondents, 447 (89.4%) completed all the questionnaire items related to TPB constructs. Review of missing patterns data did not reveal that missing variables or items were attributed to certain IDs or variables; therefore, data were not imputed for missing values. After review of the distribution of the demographic variables, income was collapsed into two categories: Less than $50,000 (Reference) and greater than or equal to $50,000.

## 3. Results

### 3.1. Qualitative Study Findings 

Twenty-six gardeners representing community gardens in Cobb (23%), DeKalb (30.8%), Fulton (30.8%), and Gwinnett (15.4%) Atlanta-metropolitan counties participated in one of five focus groups from February to March 2017. Focus groups ranged in size from four to eight participants, and the focus group discussions ranged from 75 to 90 min. Most focus group participants were female (76.9%) and identified as non-Hispanic/Latino (88.5%), white (69.2%), or black (26.9%). Most of the focus group participants were 35 years or older (84%; 40% between the ages of 36–55) and had at least some college education (96%). Regarding their community gardens’ soil, 58% indicated that soil had been previously tested. Of the 14 participants who had previously tested their soil, only one had tested for heavy metals, 13 (86.7%) had tested pH, and 15 (88.2%) had tested for nutrient content (e.g. phosphorus, potassium). 

Illustrative quotes demonstrating key themes are given in Appendix A. When describing their community garden, focus group participants frequently expressed that the garden provided benefits such as positive engagement of different generations, economic backgrounds, and vulnerable populations (e.g., handicapped, ex-offenders). In addition to community engagement, other community garden benefits mentioned were donating produce to food insecure communities, promoting environmental stewardship and awareness even outside of the garden, serving as a great educational tool for children, and facilitating a spirit of community. 

Participants described where they received information about various hazards while gardening. The most frequently mentioned sources were from other fellow gardeners and farmers (particularly peers from training programs), followed by the Extension Office. Other information sources included university websites, social media, online forums, internet searches, and health departments. Participants shared that events (e.g., training activities, workshops) and information (e.g., newsletters, flyers) provided by their community garden were very influential. Participants were asked to discuss types of risks encountered while gardening, and the most frequently reported risks were physical which included pests (e.g., fire ants, insects, snakes), proper tool handing, heat stress/proper hydration, broken glass and other debris. Ergonomics and proper tool usage were cited as safety factors. Security concerns such as being alone in the garden, theft, and vandalism were often mentioned. Participants also mentioned biological hazards such as Mosaic virus, fungus, and pet waste. Most participants indicated that chemical hazards are not a strong concern compared to other hazards. These participants stressed that most gardeners think that gardening benefits outweighed any potential risks. Of the participants that expressed concerns about chemical hazards, they were most often associated with previous site use, chemical spraying, wood chips, flooding, and water pollution. 

A few participants expressed that they had thorough knowledge about previous activities that had taken place on their garden land and used Sanborn maps (fire insurance maps that provide building purpose and layout) to identify previous owners/site use. However, other gardeners did not know or were unsure about previous site use, and they expressed concerns about historical industrial activity, dumping of gasoline and other hazardous materials, flooding, and downstream run-off. One gardener expressed that some gardeners may be more vigilant about current hazards than past hazards.

Advantages of soil testing included early identification of soil quality problems, ability to take appropriate actions in advance, and peace of mind. Other advantages were verifying soil quality from purchased or donated soil, satisfying soil certification needs (e.g., USDA organic, natural), and saving time and money on soil treatment and amendments. Primary soil testing disadvantages were concerns about liability if high levels of contaminants were discovered and associated costs (e.g. remediation, soil replacement, closing the garden), fear of scaring people away from gardening, and perceived lack of need. Several gardeners shared that raised beds may preclude the need for soil testing. The time and resources needed to collect, ship, and receive sample results were discussed as disadvantages. Additionally, some gardeners expressed concerns about the accuracy, specificity, and sensitivity of the soil test results and soil sampling techniques. 

Participants stated that supporters of soil testing would be environmental groups (e.g., Sierra Club, Kiwanis Club), other gardeners, Extension Service/Master gardeners, Parks Department, school administrators, parents, government agencies, and gardening experts. Participants also mentioned that gardening training programs and food advocacy groups would be strong supporters of heavy metal soil testing. Participants shared that critics of soil testing would be chemical companies, landowners, stakeholders concerned about property values, and experienced gardeners who are aware of garden management practices and site history. 

Facilitators of soil testing were lowering costs, increasing accessibility, desire to understand soil quality, and increased educational outreach on where to send samples, how to collect samples, and interpret soil testing results. Participants suggested that clearer language regarding the soil testing results, level of uncertainty about the results, and guidance on next steps after testing would encourage gardeners to test their soil. To facilitate soil testing, participants also recommended (1) Gardening training should include a stronger emphasis on soil testing and site history knowledge; (2) Supplies to collect and ship samples should be available at the gardening site; and (3) Volunteer organizations should collect and analyze the samples. Soil testing barriers included cost, time, accessibility, and lack of knowledge regarding where to get samples tested, how to collect samples, and how to interpret the results. 

### 3.2. Quantitative Study Results

#### 3.2.1. Participant Demographics and Community Garden Characteristics

As shown in Table 2, half of the respondents were aged 36–55 years, with only 4.0% older than 66 years old. Seventy four percent of the questionnaire respondents were male, and two percent of participants identified as Hispanic/Latino. The majority of respondents were White (87%), followed by Black or African American (4.8%) and American Indian or Alaska Native (3.2%). Vocational/Technical School was the most common level of education (58%), followed by some college /college degree (22.3%). Most respondents reported their household income to be $50,000 to $99,000 (74%). After dichotomizing the income variable, most participants reported incomes as $50,000 and above (380, 83%).

The questionnaire data included responses representing community gardens from all 50 U.S. States. The states with the highest number of respondents were California (8.7%), Massachusetts (8.5%) and Washington (7.4%). Most participants were affiliated with gardens in the South (30.49%) and the Northeast (27.5%) (Table 1). The most frequently reported types of community gardens were in park (34.6%), neighborhood (34.0%) and school settings (9.0%). Over half of the participants indicated that their community garden was located on at least 0.5 acres of land or more.

Thirty-three percent of participants reported that their garden had been established for 1–5 years, but almost a third (31.3%) did not know how long the garden had been in existence. About half of participants reported garden site history as a park site, 10% as a vacant lot, 9.4% industrial site and 16.7% didn’t know the site history. Only 11% of respondents reported that they used conventional methods (involving pesticides) in their community garden and over half of participants indicated that children are often present in the garden.

In terms of potential hazards that could contribute to soil contamination, 6% of participants indicated that their gardens were in close proximity (less than 3 feet) to roadways, and 49% of participants were unsure whether their garden was near pre-1978 housing. Only 18% of participants reported that they had not conducted a heavy metal soil test at their garden plot within the past 3 years. About 40% of the participants indicated that they had concerns that gardeners may be exposed to heavy metal contaminants through community gardens.

#### 3.2.2. Description of TPB Constructs

Study participants reported positive attitudes for soil testing (M = 3.95, SD = 0.76) (Table 3). Mean scores for subjective norms were closer to neutral (M = 3.35, SD = 0.96). The average score for “I feel social pressure to conduct heavy metal soil testing in my community garden plot” was 2.72 on a scale of 5, indicating relatively low perceived social pressure influence. Soil testing PBC items ranged from neutral to low, with the lowest scored item related to whether conducting heavy metal soil testing was up to the gardener (M = 2.52, SD = 1.01). Most participants exhibited strong intentions to conduct heavy metal soil testing with 78% exhibiting a high intention for soil testing. The internal consistency of the TPB constructs were acceptable (Cronbach alpha > 0.6).

#### 3.2.3. Logistic Regression

The adjusted logistic regression models explained 57% of the variance (Adjusted R^2^ = 0.57) of the intention to test soil (Table 4). TPB variables accounted for 34% of soil testing intention. Variables that were significantly associated with intention to test soil, in bivariate analyses, were past soil testing behavior, garden context, income, age, race, education, garden region, gardener chemical practice method and garden site history. These variables were therefore adjusted for in the logistic regression model. All TPB variables were statistically significant in the soil testing models. The odds of soil testing intention increased with a positive attitude (aOR = 2.46, 95% CI: 1.34, 4.53), stronger subjective norms (aOR = 3.39 95% CI: 2.07, 5.57), and higher PBC (aOR = 1.81, 95% CI: 1.10, 2.99). Education and income also had a statistically significant influence on soil testing intention. Gardeners with some college to completion of college education (OR= 0.11 95% CI: 0.03,0.41) and graduate school or higher (OR = 0.05, 95% CI: 0.01, 0.21) were significantly *less* likely to have a higher intention to test their soil compared to gardeners with a vocational/technical school training. The odds of soil testing intention increase by a factor of 4.00 for gardeners that have incomes greater than $50,000 compared to gardeners that have incomes less than $50,000, holding other variables constant.

## 4. Discussion

Findings from this mixed methods study illustrate that the TPB serves as a relevant framework for examining how community gardeners decide whether to test their soil for heavy metals. To our knowledge, this is the first study to utilize the TPB to measure factors that influence heavy metal soil testing intentions among a large sample of community gardeners across the United States. The quantitative component of the study was preceded by elicitation of experiences and beliefs regarding garden hazards and heavy metal soil testing through focus groups with community garden leaders in Atlanta. Additionally, the underlying gardening motivations, risk perceptions, and site history themes were examined to provide contextual data as to why some gardeners test while others do not. The combination of qualitative and quantitative approaches provides a more holistic understanding of the research question than either approach individually [50].

Questionnaire results indicate that the intention to test soil was significantly associated with attitude, subjective norms, and PBC. These variables accounted for 34% of the overall variance in soil testing in the unadjusted logistic regression models. These results are comparable to a metanalysis of TPB studies, which illustrated that the TPB variables account for an estimated 39% variance of intention across a range of behaviors [51]. Most of the study participants indicated that they intended to test their soil for heavy metals within the next growing season or sometime next year. Given these strong behavioral intentions, investigation of factors that underlie intentions become more critical to facilitate transition from behavioral intention to action. 

Questionnaire participants reported positive attitudes towards heavy metal soil testing, with most participants agreeing that heavy metal soil testing is important, beneficial, and useful. Similarly, behavioral belief findings from the focus groups suggest that gardeners’ value heavy metal soil testing as a method to improve the soil quality and grow healthy food; however, primary disadvantages were related to perceived lack of need, liability, garden stigma concerns, and amount of time to collect, ship, and analyze soil samples. Due to having raised beds with imported soils, several focus group participants expressed that their soil did not need to be tested. Installation of raised beds and filling of beds with clean/tested materials may reduce some potential soil contaminant exposures; however, raised beds cannot prevent windblown dust or other airborne contaminants, particularly if gardens are located near heavily trafficked roadways [24]. 

Questionnaire results indicated that gardeners agree that influential people would want them to test their garden’s soil; however, most participants reported that they did not feel social pressure to test. Focus groups revealed that normative beliefs or potential social pressures to conduct soil testing were from gardening peers, government, university, and relevant advocacy organizations. Focus group participants also shared that they received most of their information about gardening hazards from their gardening peers, Extension Service, and non-profit organizations. Information sources for gardening hazards included university websites, social media, online forums, internet searches, and their community garden training events and outreach activities. These findings provide potential outreach methods, information sources, and advocates to help encourage soil testing among community gardeners.

While questionnaire participants reported favorable attitudes and influential subjective norms towards soil testing, participants ranked PBC for soil testing neutral or low, indicating that gardeners may experience barriers related to heavy metal soil testing. Focus group participants expressed that control beliefs and barriers to soil testing were centered around liability, costs, accessibility, and concerns related to sample representativeness and results interpretation. For example, mandatory disclosure of soil lead levels for real estate properties and other regulations may deter gardeners from heavy metal soil testing [52] due to potential consequences such as stigma, decreased property values, and required clean up. These study results support the findings of other site-specific research of community gardeners, which concluded that soil testing challenges were related to perceived behavioral control challenges such as paucity of training, insufficient financial support, and difficulty interpreting results [13,27,30,32]. Similar to other studies of food safety and protective behaviors, targeting PBC, particularly related to self-efficacy in interventions may help to increase behavioral compliance [53,54,55]. Moreover, studies recommend that high PBC to perform a behavior occurs when people: (1) believe they can perform the behavior; (2) possess the resources to conduct the behavior; and (3) are able to overcome or manage barriers to the behavior [35,56].

The quantitative analysis also investigated demographic factors and garden characteristics that may influence soil contaminant levels and intention to conduct a soil test. While geographic region, age, race, garden site history, chemical methods, and past soil testing behavior were not significant, income and education level were significant covariates. Higher income was associated with soil testing intention. Since heavy metal soil testing can be expensive, those with higher incomes may be more likely to afford testing. Surprisingly, participants with college education or higher were less likely to intend to soil test than those with vocational/technical school training. Although we cannot explore this in our study, it may be that those with vo-tech degrees were more likely to have horticultural training that may include soil quality and testing metrics. 

Garden site history and other contextual information provide insight into factors that could contribute to elevated contaminant concentrations in the soil. Questionnaire data indicates the gardeners have varied knowledge and beliefs about contaminant risk factors. About half of questionnaire participants did not know whether their garden was near older housing that may have lead paint, and about 31% did not know how long their garden had been established. However, most participants had some awareness of their garden’s previous site use, and less than half were concerned that community gardeners may be exposed to heavy metal contaminants. The qualitative data supports quantitative findings regarding diverse perspectives and recognition of gardening risks. When focus group participants discussed gardening hazards, chemical contaminants were not at the forefront of their concerns. Similar to another study, physical hazards such as theft, ergonomics, and pests were more commonly mentioned [29]. Qualitative findings revealed that gardening benefits and motivations were community engagement, cultural identity, environmental stewardship, food creation, and children’s education [1], and these advantages prevailed over any perceived risks. Some focus group participants who expressed concerns about past dumping and other land use activities at their garden site shared that they lacked information on how to confirm garden site history. 

These findings have meaningful implications for outreach regarding gardening risks and safety interventions. First, focus group findings suggest that emphasis on soil health/quality to grow healthy food may strongly resonate with gardeners. Comparable to other studies, these findings suggest that framing the positive outcomes of soil testing may motivate gardeners more than a problem identification approach [28]. Second, this study identified referents (e.g. environmental groups, gardening training programs) and information sources that could influence some gardeners’ perceptions of gardening hazards and soil testing. Through targeted educational outreach, these referents and information sources can help increase awareness of soil contamination risks, site history, and soil testing. For example, county extension offices and garden stakeholders can provide a checklist of garden location considerations that include investigation of the site history, inventory of potential current hazards (e.g., proximity to roadways, older housing) and local laboratories that conduct heavy metal soil testing [57]. 

Next, the study results illustrate potential opportunities to reduce identified barriers to heavy metal soil testing. Development of rapid, low-cost soil testing tools [29,58,59] and partnership programs to make soil testing more accessible may influence some gardeners to test their soil. For example, universities, public health departments, citizen-scientists, and other organizations can collect and test soil samples, educate gardeners about the soil testing results, and provide training resources on gardening hazards [60,61,62,63]. Study findings also warrant development on clear, consistent guidelines regarding threshold levels for garden soil contaminants as well as bioavailability assessments to quantify the extent that contaminants are absorbed in human tissue. Since advisory guidelines for garden soil contaminants vary by jurisdiction, local gardening stakeholders should consider providing advice on next steps if contaminant levels are high. Next steps include counsel on behaviors to reduce contaminant exposure (e.g., handwashing), funding for soil remediation options, and support to handle any property disclosure ramifications. Technical assistance with these and other follow up actions may address gardeners’ uncertainties, concerns, and perceived consequences of soil testing. 

Exposures to chemicals found in soil contaminants can have long term effects among children and other vulnerable populations even at low dose exposures [64,65]. The importance of modeling and enforcing safe gardening behaviors is underscored by of the majority of questionnaire participants indicating that children either sometimes or frequently visit their community garden and some participants indicating that their garden was in a school context. Research indicates the number of U.S. school-based gardens has increased over time, particularly among schools with a USDA Farm to School based program [66]. Since the Community Preventive Service Task Force as well as international organizations recommend school gardening interventions to increase vegetable consumption among children [67,68], the number of school and early childhood education gardens may continue to increase. Moreover, school gardens have been implemented as an environmental equity tool in minority and low-income settings where industrial emissions and other environmental hazards may be more prevalent [69,70]. Incorporating soil testing as well as other good agricultural and handling practices can reduce potential environmental hazards and liability concerns for school garden stakeholders [71,72]. 

There are several strengths to this study. By incorporating theory-based questionnaire items derived from focus group formative research, behavioral determinants of soil testing could be explored and potentially targeted for behavioral intervention. This study incorporated a relatively large and geographically diverse sample of community gardeners in the United States. Previous studies of community gardens are often city or region specific; therefore, challenges arise in comparing practices among different cities. Additionally, this study adds to the literature by inclusion of community gardens in different contexts (e.g. parks, schools, neighborhoods), chemical-practice methods, site histories, and garden proximity to roads and older housing. Garden contextual information can give rise to how specific chemical practices and garden siting policies could be better integrated into training to promote safe behaviors and awareness. These contextual factors may influence concentrations of soil contaminants and may affect other gardening behaviors. For example, one study has suggested that more well-established gardens that implemented soil tilling over long period of time may have resulted in dilution of soil contaminants and lower soil contaminant concentrations [32].

Despite these strengths, this study has several limitations that should be taken into consideration. Our participants may represent a more self-motivated subset of our population with a higher affinity to health–related topics, and consequently, have generally different sentiments regarding the study questions than those who did not participate. Secondly, focus groups may have the potential to invoke information bias if the group is swayed by the moderator or if there’s deference to perceived dominant participants. For example, some participants may have had deference to the Extension agents and Master Gardeners in the focus groups. Additionally, social desirability bias can occur when focus group participants give responses that they believe the group feels are acceptable instead of responses based on their true feelings and experience. The soil screening incentive for focus group participants may have inadvertently influenced participants’ responses regarding soil testing and impacted their decision to participate in the study.

The questionnaire utilized a cross-sectional design; therefore, the study was unable to access causality between soil testing intentions and future soil testing behavior. While intention is typically a strong predictor of behavior, it is not a surrogate for actual behavioral execution. Self-reported soil testing history was accounted for in the analysis, but records were not obtained to verify whether participants had previously tested their soil. The study examined injunctive norms (others’ expectations) for subjective norm measurement as specified by TPB, but did not assess descriptive norms (others’ behavior) which may also influence intention [73]. Additionally, most study participants were white males and may not be representative of the population of community gardeners in the United States. Convenience sampling, particularly through recruiting members of the ACGA and Facebook groups, presents a social connectivity among participants who may share similar gardening convictions that may not be reflective of community gardeners outside this social circle. Finally, individual behavior is multi-factorial, and constructs not explicitly included in the present study such as knowledge, trust, and risk perception, may interact with TPB variables to further explain behavioral intention [74,75,76]. 

## 5. Conclusions

Community gardeners in different contexts experience multiple, competing challenges that require time and resources [77,78]; therefore, interventions to address safe gardening practices need to be practical and efficient. This study makes a unique contribution by providing a theoretical framework to prioritize specific intervention targets that predict soil testing intention. Public health interventions founded in theoretical frameworks have been shown to be more effective in modifying behavior than those not using theory [79]. 

Several studies have outlined challenges that gardeners may face when confronting soil contamination testing and sustainable practices for urban soil management [80]; however, fewer studies have examined underlying factors to improve behavioral interventions that address these challenges. The study results suggest that theory-based interventions targeted on improving attitude, subjective norms, and PBC may influence soil testing. Future studies could examine pre- and post-interventions focused on training and soil testing assistance. Community gardening training should not only be aimed at improving soil contaminant knowledge but should also address TPB variables such as perceived behavioral control of soil testing [81,82]. It should be noted that TPB is an individual behavioral model that does not directly address the complex, multi-level factors that affect community garden soil management. For example, land use policies and environmental justice concerns related to vacant lands provide context for potential soil pollutants and should be explored further in research related to community garden soil testing and best management practices [28,83].

## Figures and Tables

**Table 1:** Community Garden characteristics reported by respondents.

Garden Characteristic/Perspective		*n*	Percent
Garden Context	Faith-based	23	4.9
	Government Facility	7	1.5
	Healthcare Facility	16	3.4
	Neighborhood	161	34.0
	Other	25	5.3
	Park	164	34.6
	School	43	9.1
	Senior Center	35	7.4
Region	Midwest	72	15.4
	Northeast	129	27.5
	South	143	30.5
	West	125	26.7
Urban Classification	Other	5	1.1
	Rural	24	5.1
	Suburban	202	42.8
	Urban	241	51.0
Garden Years of Operation	1–5 years	157	33.4
	6–10 years	55	11.7
	Greater than 10 years	95	20.2
	Less than year	16	3.4
	Not sure	147	31.3
Number of Gardeners	Less than 5	15	3.3
	5 to 15	297	62.9
	16 to 30	85	18.0
	Greater than 30	60	12.7
	Less than 5	15	3.2
	Not sure	15	3.2
Garden Size	Less than 0.25 acre	49	10.4
	0.24–0.49 acres	65	13.8
	0.5–0.99 acres	185	39.3
	Greater than 1 acre	172	36.5
Garden Site History	Vacant Lot	48	10.2
	Park	228	48.5
	Former Industrial Site	44	9.4
	Parking lot	15	3.1
	Farm	20	4.3
	Other	37	7.9
	Don’t know	78	16.6
Proximity of garden to a roadway/street	Less than 3 feet	30	6.5
	3–9 Feet	365	78.5
	Greater than 9 feet	70	15.0
Older (pre-1978) housing/buildings near garden	Yes	63	13.4
	No	176	37.5
	Not Sure	231	49.1
Gardening Chemical Practices	Conventional (With pesticides)	52	11.0
	Natural (without known use of pesticides)	253	53.6
	USDA Certified Organic	153	32.4
	Other	14	3.0
Allow Children in the Garden	Never	8	1.7
	Rarely	40	8.5
	Sometimes	54	11.5
	Often	288	61.2
	Not sure	81	17.2
Conducted a heavy metal soil test at garden plot within the past 1–3 years	Never	89	18.4
	At Least Once	214	44.3
	Several Times A Year	163	33.8
	Don’t Know	17	3.5
Concerned that gardeners may be exposed to heavy metal contaminants	Strongly Disagree	25	5.1
	Disagree	180	37.1
	Neither agree or disagree	83	17.1
	Agree	89	18.4
	Strongly agree	108	22.3

**Table 2:** Demographic characteristics of the questionnaire respondents.

Demographic Variable		*n*	Percent
Gender	Female	124	26.4
	Male	345	73.6
Age	18–35	179	38.0
	36–55	236	50.1
	56–65	37	7.9
	66 or older	19	4.0
Income	Less than $24,999	30	6.3
	$25,000 to $49,999	45	9.6
	$50,000 to $99,999	348	73.9
	$100,000 or more	32	6.8
	Decline to state	16	3.4
Education	Less than High School to High school Graduate	10	2.1
	Vocational/Technical School	273	57.9
	Some College to College Graduate	105	22.2
	Graduate Degree or Higher	83	17.6
Ethnicity	Hispanic/Latino	9	2.0
	Not Hispanic/Latino	453	98.0
Race	American Indian or Alaska Native	15	3.2
	Asian	9	1.9
	Bi-racial	2	0.4
	Black or African American	22	4.7
	Native Hawaiian or Other Pacific Islander	11	2.4
	White	405	87.3

**Table 3:** Theory of Planned Behavior Constructs for Soil Testing.

Construct	Soil Test Item	Mean (SD)	Cronbach’s Alpha
Attitude ^1^	To conduct heavy metal soil testing at my garden during the next growing season would be unimportant-important	4.1(0.9)	
	To conduct heavy metal soil testing at my garden within the next growing season would be harmful-beneficial	4.1(0.6)	
	To conduct heavy metal soil testing at my garden during the next growing season would be worthless-useful	3.7(1.3)	
	Overall Attitude	3.9 (0.8)	0.61
Subjective Norms ^2^	People who are influential in my garden think that I should conduct heavy metal soil testing in my community garden plot	3.8(1.1)	
	I am expected to conduct heavy metal soil testing in my community garden plot	3.5(1.4)	
	I feel social pressure to conduct heavy metal soil testing in my community garden plot	2.7(1.2)	
	Overall Subjective Norms	3.4(0.9)	0.68
Perceived Behavioral Control (PBC) ^3^	I am confident that I could conduct heavy soil testing in my community garden if I wanted to	3.9(0.8)	
	Heavy metal soil testing in my garden plot is not under my control- under my control	3.1(1.2)	
	Heavy metal soil testing in my garden plot is very difficult-very easy	3.1(1.0)	
	Whether I conduct heavy metal soil testing at my garden plot is entirely up to me	2.5(1.0)	
	Overall PBC	3.4(0.8)	0.65
Intention ^4^	I expect to conduct heavy metal soil testing at my garden plot in the future	3.7(0.88)	
	I am determined to conduct heavy metal soil testing at my garden plot in the future	3.6(0.91)	
	Overall Intention	3.7(0.4)	0.83
		*n* (%)	
	Low Intention	105(22.0)	
	High Intention	372(78.0)	

^1^ 1 = negative attitude (e.g., unimportant, harmful); 5 = positive attitude (e.g., important, beneficial); ^2^ 1 = low subjective norm influence (strongly disagree); 5 high subjective norm influence (strongly agree); ^3^ 1 = low pbc (e.g., strongly disagree, difficult) 2 = high pbc (e.g., strongly agree, easy); ^4^ 1–3.5 = low intention (strongly disagree); >3.5 = high intention (strongly agree).

**Table 4:** Predictors of Soil Testing Intention.

Predictor	OR ^a^	[95% Conf. Interval]
Attitude	2.46 *	[1.34,4.53]
Subjective Norms	3.39 *	[2.07,5.57]
Perceived Behavioral Control	1.81 *	[1.10,2.99]
Past Testing Behavior	1.15	[0.85,1.57]
Region	0.36	[0.11,1.18]
Income	4.00 *	[1.52,10.51]
Age	0.76	[0.47,1.21]
Race	0.81	[0.40,1.67]
Education (Reference: Vocational School)		
Less than High School to High School/GED Graduate	0.95	[0.11,7.85]
Some College to Completion of College	0.11 *	[0.03,0.41]
Graduate School or Higher	0.05 *	[0.01,0.21]
Chemical Practice Methods	0.66	[0.01,1.05]
Garden Site History	1.21	[0.98,1.50]
pseudo R^2^	0.57	

* *p* < 0.05, ^a^ Adjusted for all covariates in table; Logistic regression models were fit using robust standard errors. For all contextual variables (i.e., garden context, income, region, age, race, education, garden site history), the reference value was set at the most frequent category.

## Appendix A

**Table A1:** Quotes Representing Focus Group Themes.

Theme	Representative Quotes
Community Garden Benefits	“We have everything you can imagine in our neighborhood, including USDA food desert, although we do have a gated community as well…gardening is such a great way to bring people together from all different cultures and socioeconomic levels.… we have people from all sections of the community that come and garden together” “I’m trying to retrain my people, meaning folks that look like me, Black Americans, to get back into that village mentality where you just take what you need and leave for others, and share in the work…because we’ve been put into a society that’s very individualistic, and that’s been harmful for our communities to become individualistic, because we’re not looking out for each other” “[Community gardening] increases their awareness of the relationship to the Earth, and I think it promotes recycling amongst our gardeners, like in their home life … it just brings it to their awareness and makes them more prone to recycle and reuse”
Gardening Hazards and Risk Perception	“We do have a number of potential hazards…sharp items like tools, bee stings, fleas, spiders, fire ants, mosquitoes…Vandalism or people unknown walking into the garden when someone is alone. That’s a concern that we have a sensitivity about.” “I don’t think they worry about heavy metals…they’re interested in what do I need to add to make this soil healthier so I can grow vegetables.” “I think there are some people who feel that it would test everything, we worry too much about everything, and end up spending money on things rather than just getting on with it.”
Site History	“… I don’t think that most people are looking like at the entire land history” “They think a lot about what they or the other gardeners could put on the site, but they don’t think about what’s already on the site.” “So prior to that it may have been a farm, I’m not sure. I just worry more about things that could have been dumped there” “those were old farm sites and people don’t think about the arsenic that was in the soil…and it doesn’t go away. It’s there a long time.”
Behavioral Beliefs/Attitude	“I think if you know what’s there, it lets you know if there’s a problem that you might want to deal with in terms of heavy metals and pesticides. If you know what’s there in terms of the nutrients, then you’ve saved yourself money by not over fertilizing.” “And then there’s the added thing of you know when your soil is out of whack and your vegetables aren’t growing right…so you avoid the problem by starting out right with knowing what’s in there.” “I think it might cause some fear in your everyday gardener to not rent a bed if they felt like there was danger that it had toxins or metals in it”
Normative Beliefs/Subjective Norms	Non-supporters of soil testing: “Companies that have to get rid of stuff.”; “Yeah. They’re dumping, and they know they’re dumping.” “I don’t know, I imagine it (contaminants) would have to be in really high concentrations, but anybody interested in property values.”
Control belief/Perceived Behavioral Control	“But one more thing about encouraging this sampling, you could have sort of a formal thing. Anybody who wants to do it, we’ll all do it together at the same time during the work day, and then somebody can take the samples over.” “They give you parts per million and a lot of times there’s not a definite level that’s acceptable, because nobody really knows how much is acceptable, so they’ll say it’s EPA limits or below EPA limits or something, but you still don’t know if that’s safe.” “…but you’ve got to be careful not to scare and upset people unnecessarily, because sometimes these things with the environment get blown way out of proportion.”
